# Mmu-miR-125b overexpression suppresses NO production in activated macrophages by targeting eEF2K and CCNA2

**DOI:** 10.1186/s12885-016-2288-z

**Published:** 2016-03-28

**Authors:** Zhenbiao Xu, Lianmei Zhao, Xin Yang, Sisi Ma, Yehua Ge, Yanxin Liu, Shilian Liu, Juan Shi, Dexian Zheng

**Affiliations:** State Key Laboratory of Medical Molecular Biology, Institute of Basic Medical Sciences, Chinese Academy of Medical Sciences & Peking Union Medical College, Beijing, 100005 China

**Keywords:** Mmu-miR-125, Macrophages, Nitric oxide, eEF2K, CCNA2

## Abstract

**Background:**

MicroRNAs have been shown to be important regulators of the immune response and the development of the immune system. It was reported that microRNA-125b (miR-125b) was down-regulated in macrophages challenged with endotoxin. However, little is known about the function and mechanism of action of miR-125b in macrophage activation. Macrophages use L-arginine to synthesize nitric oxide (NO) through inducible NO synthase (iNOS), and the released NO contributes to the tumoricidal activity of macrophages.

**Methods:**

Luciferase reporter assays were employed to validate regulation of a putative target of miR-125b. The effect of miR-125b on endogenous levels of this target were subsequently confirmed via Western blot. Quantitative reverse transcription-polymerase chain reaction (qRT-PCR) was performed to determine the expression level of miR-125b in macrophage. MTS assays were conducted to explore the impact of miR-125b overexpression on the cell viability of 4T1 cells.

**Results:**

Here, we demonstrate that mmu-miR-125b overexpression suppresses NO production in activated macrophages and that LPS-activated macrophages with overexpressed mmu-miR-125b promote 4T1 tumor cell proliferation in vitro and 4T1 tumor growth in vivo. CCNA2 and eEF2K are the direct and functional targets of mmu-miR-125b in macrophages; CCNA2 and eEF2K expression was knocked down, which mimicked the mmu-miR-125b overexpression phenotype.

**Conclusions:**

These data suggest that mmu-miR-125b decreases NO production in activated macrophages at least partially by suppressing eEF2K and CCNA2 expression.

**Electronic supplementary material:**

The online version of this article (doi:10.1186/s12885-016-2288-z) contains supplementary material, which is available to authorized users.

## Background

Macrophages are key components of the mammalian innate immune system, in which they function in cytokine release, pathogen killing and antigen presentation to the adaptive immune system. When cell surface sensing proteins, such as Toll-like receptors (TLRs), recognize and engage pathogens, macrophages are rapidly activated; these activated macrophages transform from a relative quiescent state to an effector state to perform defense functions [1–5]. Classically activated macrophages, or M1 macrophages, activate the Th1 immune response and secrete high amounts of pro-inflammatory mediators, such as cytotoxic TNFα and nitric oxide (NO), to kill invading pathogens or tumor cells. In fact, the high expression of inducible NO synthase (iNOS), which produces NO, is the hallmark of these macrophages. NO, a free radical gaseous molecule, is a mediator of vital physiological functions, including host defense. Many cell types can produce NO using L-arginine via iNOS. Macrophages are one of the best-characterized sources of NO. Throughout the last decade, NO has been identified to play an important role as a first line of defense against various pathogens. Macrophage uses L-arginine to synthesize NO via iNOS, and the released NO contributes to the tumoricidal activity of macrophages. In early stages of tumor development, macrophages employ their killing mechanisms, particularly the generation of high NO concentrations, to induce tumor cell apoptosis and destroy emerging transformed cells [6–8].

It has been shown that microRNAs (miRs) are important mediators of macrophage activation. It was reported that miR-155, miR-146, miR-147, miR-9, miR-107 and miR-21 are induced by the TLR signaling pathway [9–13]. These miRs can inhibit the expression of signaling proteins in the inflammatory signaling cascade and therefore modulate immunity through feedback mechanisms [10, 12]. MiR-125b, a homolog of *C. elegans* miR-lin-4, is deregulated in most cancers and can regulate cancer cell proliferation via its target genes [14–19]. It has also been demonstrated that miR-125b is down-regulated in macrophages in response to TLR4 signaling [20–24] and enriched in hematopoietic stem cells, which then enhances hematopoietic engraftment [25, 26]. The mechanisms by which macrophages respond to miR-125b and the function of miR-125b in regulating macrophages remain unclear.

In the present study, we demonstrate that mmu-miR-125b (MIMAT0000136) is down-regulated in macrophages activated by LPS. Mmu-miR-125b over-expression inhibits NO production and thus promotes cancer cell growth both in vitro and in vivo. We further determined that eEF2K and CCNA2 are the important target genes of mmu-miR-125b in macrophages. Knockdown of eEF2K and CCNA2 expression mimics the phenotype of mmu-miR-125b overexpression in macrophages. These data suggest that mmu-miR-125b decreases NO production in activated macrophages to promote cancer cell growth, at least partially by suppressing eEF2K and CCNA2 expression.

## Methods

### Isolation of peritoneal macrophage and cell cultivation

Mice were injected intraperitoneally (i. p.) with 2 mL of 3 % thioglycollate (Difco, Detroit, MI, USA). Three days later, mice were sacrificed by CO_2_ inhalation followed by cervical dislocation. Peritoneal exudate cells were enriched for the peritoneal macrophages using the method as described by Kumagai et al [27]. Briefly, the peritoneal cells were harvested by lavage and washed for three times with the complete culture medium. Approximately, 1 × 10^6^ cells per well were then cultured for two hours in six-well plates allowing the macrophages to adherent. The cells were washed three times with warm Hank’s balanced salt solution to remove nonadhesive cells. The adherent macrophages were stimulated with various concentrations of stimuli and cultured at 37 °C with 5 % CO_2_ in DMEM or PRMI-1640 supplemented with 10 % FBS, 100 U/ml penicillin, and 100 U/ml streptomycin.

Cell lines of human HEK293T, mouse macrophage RAW264.7 and breast cancer 4T1 originated from the American Type Culture Collection (Rockville, MD). These cells were cultured at 37 °C with 5 % CO_2_ in DMEM or PRMI-1640 supplemented with 10 % FBS, 100 U/ml penicillin, and 100 U/ml streptomycin. RAW264.7 cells stably transduced with lentivirals pLL3.7-miR-125b (named as RAW264.7-miR-125b) or control empty vector pLL3.7 (named as RAW264.7-pLL3.7) were sorted by FACS. Mmu-miR-125b over-expression was verified by real-time quantitative PCR (qPCR) carried out in a step-one Real-time PCR machine (Applied Biosystems, USA).

### Quantitative real-time PCR

RNA was isolated with TRIzol (Invitrogen, USA) reagent according to the manufacturer’s instructions. qPCR was conducted using a step-one Real-time PCR machine (Applied Biosystems, USA). SYBR Green PCR Master Mix (Takara, Shiga, Japan) was used to analyze mmu-miR-125b, CCNA2 and eEF2K expression. Primer sequences are listed in Additional file 1: Table S1.

### DNA constructs

Mouse pre-miR-125b-2 gene and the 3’ UTR fragment of CCNA2 and eEF2k containing the putative mmu-miR-125b target sites and the mutations were amplified by using the specific PCR primers (The forward and reverse primers were shown in the Additional file 1: Table S1) and mouse peripheral blood lymphocyte genomic DNA as template. The DNA fragments were respectively cloned into the pLL3.7 vector (Promega, Madison, WI, USA) downstream of the U6 promoter and the psiCHECK2.2 vector (Promega, Madison, WI, USA) downstream of the renilla luciferase gene. The DNA constructs were verified with DNA sequencing by BGI Life Tech Co. Ltd. (China).

### NO detection

NO was determined using a nitrate/nitrite assay kit (Beyotime Institute of Biotechnology, China). Briefly, cells were stimulated with LPS for 12 h and the supernatants were collected by centrifugation. Concentration of NO was determined by mixing 50 μl of the supernatants with 50 μl Griess reagent I and 50 μl Griess reagent II and measured in a Multiscan ELISA Reader (Assays HiTech) at 540 nm with appropriate standards (0–60 M) and normalized by total protein concentration.

### Coculture assay

4T1 cells were cocultured with either RAW macrophage cells. Briefly, for coculture without cell-cell contact, 1 × 10^5^ LPS-activated RAW264.7-miR-125b or RAW264.7-pLL3.7 cells were seeded in Boyden Transwell inserts (0.4 μm pores; Corning) permeable for soluble factors but not cells. Transwells containing macrophages were then inserted into a 24-well plate and seeded with 3 × 10^5^ 4T1 tumor cells in each well. The cell viability of 4T1 cells was measured with MTS (3-(4, 5-dimethylthiazol-2-yl) -5-(3– carboxymethoxyphenyl)-2-(4-sulfophenyl)-2H-tetazolium)) assay according to the manufacturer’s instruction (Promega, Madison, WI) at different time points and calculated by the following formula: Viability (OD) = OD of mix well- OD of control well.

### Cell viability assays

Cell viability and growth cure was measured using MTS assay according to the manufacturer’s instruction. Briefly, the cells were seeded on 96-well plates at a density of 2 000 cells/well, incubation for indicated time, MTS solution was added (20μL/well) into the cells, and incubated for 2 h at 37 °C, followed by measuring the absorbance at 492 nm with a microplate reader.

### Animal experiments

Animal experiments were performed in accordance with the institutional guidelines for animal care and were approved by the committee for the use and care of animals of the Chinese Academy of Medical Sciences and Peking Union Medical College, Beijing, China. Briefly, 4T1 (2 × 10^6^) cells and LPS-activated RAW264.7-miR-125b or RAW264.7-pLL3.7 (5 × 10^5^) cells were subcutaneously co-injected into the right flanks of 4 to 6-week-old BALB/c female mice. Mice were closely monitored for nearly 1 month. The tumor sizes were measured every 3 days with a caliper. The tumor volume (V) was calculated using the formula: V = 0.5 × length × width^2^. At the experimental end point, animals were euthanized and tumors were removed and weighed.

### Sequence alignment

The mmu-miR-125b seed region and CCNA2, eEF2K 3’ UTR sequences from mouse (Mus musculus) were obtained and aligned using micoRNA database (http://www.microrna.org/microrna/getGeneForm.do) or Targetscan (http://www.targetscan.org/mmu_61/) [28, 29].

### Luciferase reporter assay

293T cells were co-transfected with pLL3.7-125b or pLL3.7 and psiCHECK2.2 vector containing 3’ UTRs of CCNA2, eEF2K or their mutations or miR-125b positive control. The luciferase activity was quantified after 48 h transfection using a Dual Luciferase Assay kit (Promega, Madison, WI). Firefly luciferase activity was normalized to Renilla, and the ratio of Firefly/Renilla value was reported.

### Western blot

RAW264.7-miR-125b, RAW264.7-pLL3.7 or RAW264.7 cells were lysed and total 40-60 ng proteins in loading buffer were denatured for 10 min at 95°C, and then the proteins were subjected to 10 % SDS-PAGE. The proteins in the gel were electronically transferred to an Immobilon-P membrane (Millipore, Eschborn, Germany). After blocking with 5 % no-fat milk, the membrane was incubated with a rabbit polyclonal anti-CCNA2 or anti-eEF2K or anti-GAPDH Ab (1:1000; Cell Signaling Technology, Beverly, MA) overnight in TBS. The interesting proteins were visualized using a peroxidase-conjugated anti-rabbit IgG Ab (1:10000, Cell Signaling Technology, Beverly, MA) for 1 h and detected by using ECL system (Amersham Pharmacia Biotech Europe, Freiburg, Germany) followed by exposure to an X-ray film.

### RNA interference

SiRNA used in the experiment was listed in Additional file 2: Table S2. siRNA duplexes were transfected into cells using Lipofectamine 2000 (Invitrogen) at a final concentration of 40 nM.

### Statistical analysis

All experiments were at least repeated three times. The results are presented as mean ± SD. The data were subjected to the Student’s *t-test. P* < 0.05 was considered significant.

## Results

### Mmu-miR-125b expression is down-regulated in activated macrophages

**Fig. 1:**
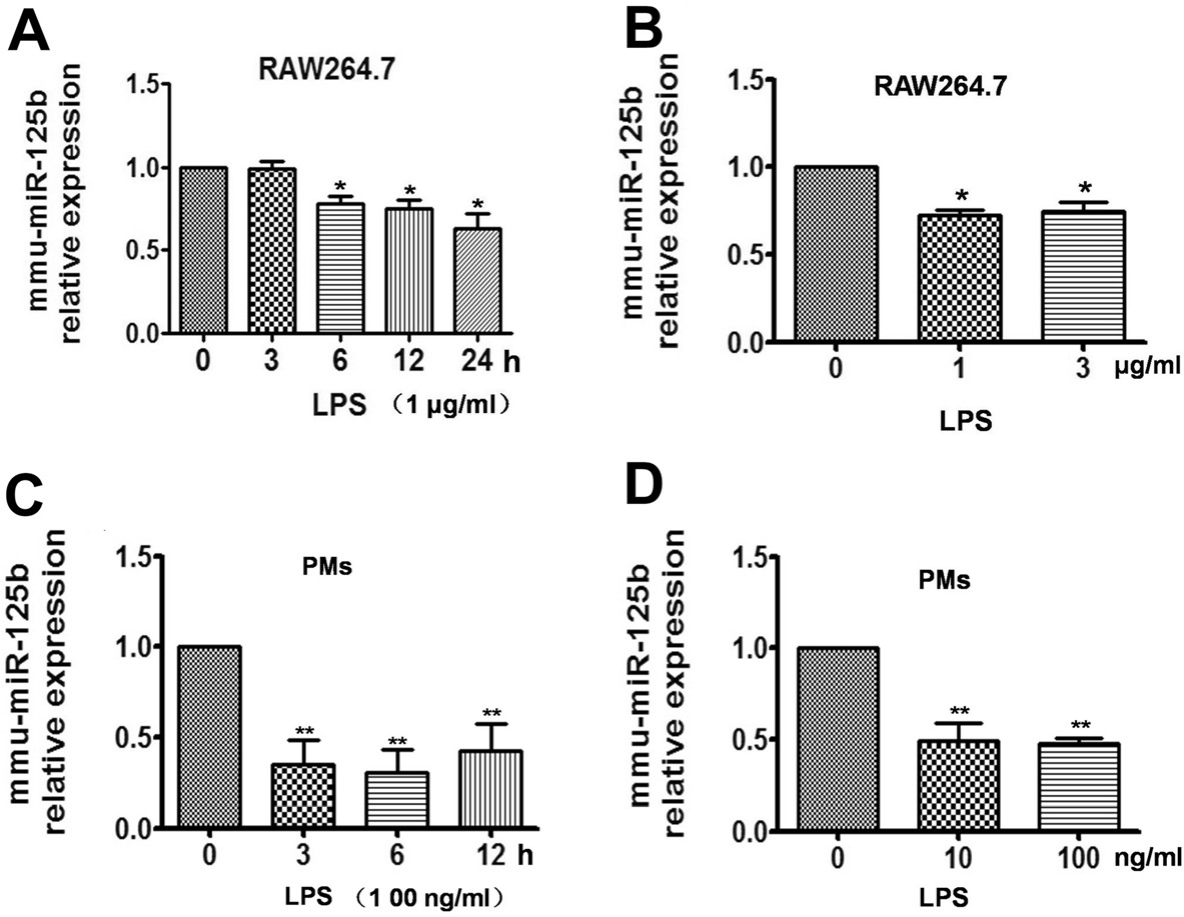
Down-regulation of mmu-miR-125b expression in LPS-activated RAW264.7 cells and peritoneal macrophages. **a** RAW264.7 cells were stimulated with 1 μg/ml LPS for the indicated time points. **b** RAW264.7 cells were stimulated with different concentrations of LPS for 6 h. **c** Peritoneal macrophages cells were stimulated with 100 ng/ml LPS for the indicated time points. **d** Peritoneal macrophages were stimulated with different concentrations of LPS for 6 h. The expression of mmu-miR-125b was determined by qPCR and normalized to the expression of U6. The data are presented as the mean ± SD (*n* = 3) of three independent experiments. **p < 0.01; *p < 0.05

MiR-125b is an important microRNA in cancer and the immune response. It has been reported that miR-125b is down-regulated in macrophages in response to TLR4 signaling [22]. However, little is known about the function and mechanism of action of miR-125b in macrophage activation. To determine the expression level of mmu-miR-125b in macrophages, mouse RAW264.7 and peritoneal macrophages were stimulated with LPS at various concentrations for different time points, and total RNA was then extracted with TRIzol. Mmu-miR-125b expression was determined by reverse transcription using a stem-loop primer (Additional file 1: Table S1) followed by SYBR Green quantitative PCR (qPCR). As shown in Fig. 1a, mmu-miR-125b expression decreased over time in RAW264.7 cells activated with 1 μg/ml LPS. Mmu-miR-125b expression was also down-regulated by different concentrations of LPS (Fig. 1b). Similar results were obtained in peritoneal macrophages (PMs) activated with LPS (Fig. 1c-d), indicating that mmu-miR-125b expression is down-regulated in macrophages activated by LPS.

### MiR-125b overexpression suppresses NO production and iNOS expression in activated macrophages

**Fig. 2:**
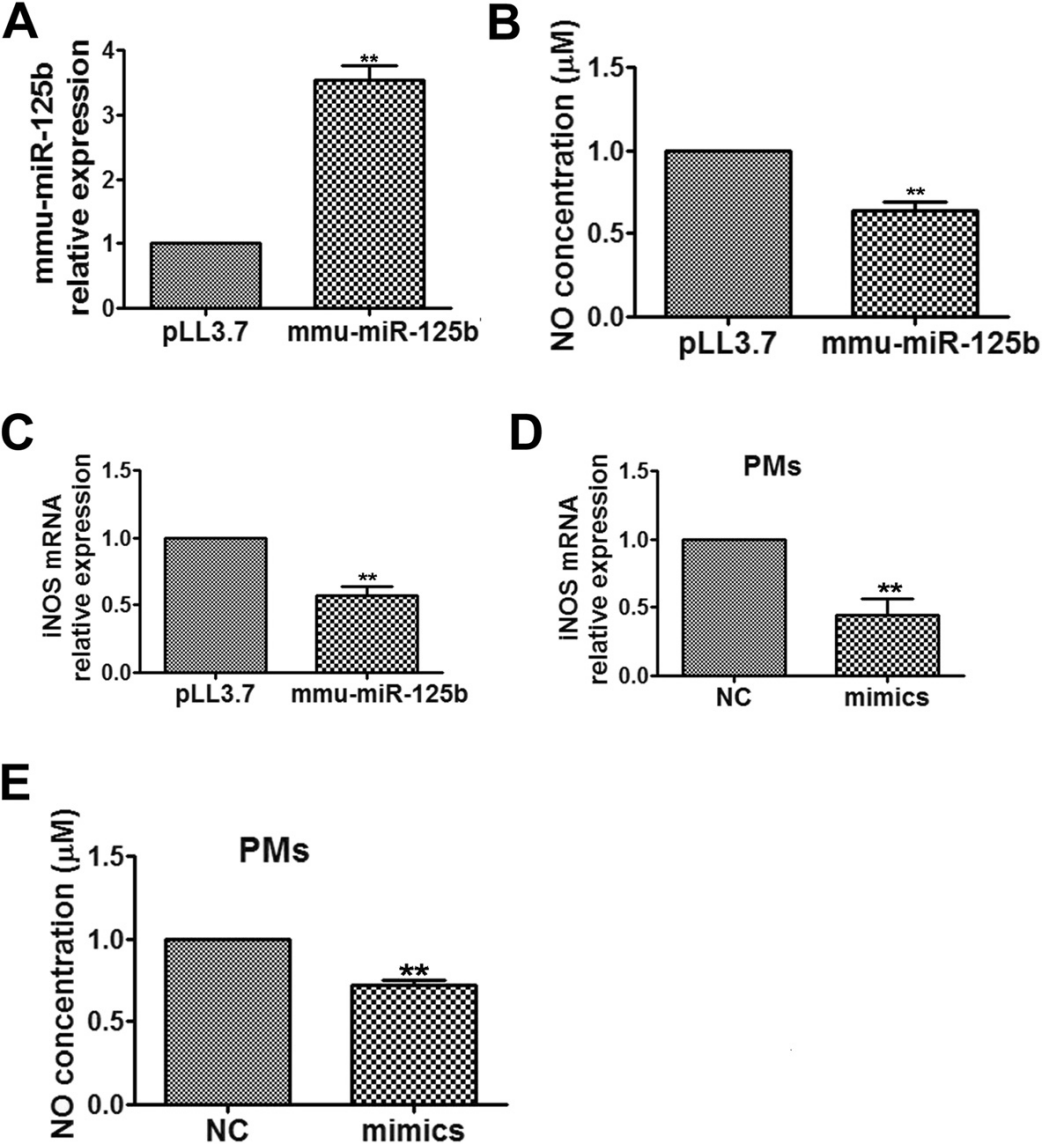
Mmu-miR-125b inhibits NO production and iNOS mRNA expression in LPS-activated RAW264.7 cells and peritoneal macrophages. **a** The relative expression of mmu-miR-125b was determined in RAW264.7 cells infected with the pLL3.7-mmu-miR-125b lentivirus and sorted by FACS for GFP expression. **b** RAW264.7 cells overexpressing mmu-miR-125b (RAW264.7-miR-125b) and control cells (RAW264.7-pLL3.7, pLL3.7) were stimulated with 1 μg/ml LPS for 6 h. The supernatants were collected to measure NO using a nitrate/nitrite assay kit and normalized to the expression of total proteins. **c** iNOS mRNA levels were measured by qPCR and normalized to the expression of β-actin. **d** Peritoneal macrophages were transfected with mmu-miR-125b mimics or controls at a final concentration of 40 nM for 24 h. The cells were stimulated with 100 ng/ml LPS for 6 h, iNOS mRNA levels were measured by qPCR and normalized to the expression of β-actin. The data are presented as the mean ± SD (n = 3) of three independent experiments. **e** The supernatants were collected to measure NO using a nitrate/nitrite assay kit and normalized to the expression of total proteins. **p < 0.01; *p < 0.5

Classically activated, or M1, macrophages are activated by TLR ligands. In fact, the high expression of iNOS, which produces NO, is the hallmark of these macrophages. NO has been shown to play an important role as a first line of defense against various pathogens. To assess the role of mmu-miR-125b in activated macrophages, NO production was evaluated in macrophages transduced with mmu-miR-125b. Recombinant lentivirus encoding mmu-miR-125b was packaged, and RAW264.7 cells were infected with the lentivirus. Cells with stable expression of mmu-miR-125b (RAW264.7-miR-125b cells) were sorted by fluorescence-activated cell sorting (FACS). As shown in Fig. 2a, mmu-miR-125b expression in RAW264.7-miR-125b cells was approximately 4-fold higher compared to that in control cells. Then, RAW264.7-miR-125b cells were activated with LPS, and the concentration of NO in the cell lysate was determined. As shown in Fig. 2b, NO production in LPS-activated RAW264.7-miR-125b cells was significantly down-regulated compared to that in control cells infected with Lenti-GFP control. Real-time qPCR demonstrated that iNOS mRNA expression was simultaneously decreased (Fig. 2c). These results on NO production and iNOS expression were confirmed in peritoneal macrophages transfected with chemically synthesized miR-125b mimics (Fig. 2d, e). These data indicate that miR-125b overexpression significantly suppresses iNOS-catalyzed NO production in LPS-activated macrophages.

### LPS-activated macrophages with miR-125b overexpression promote tumor cell proliferation

**Fig. 3:**
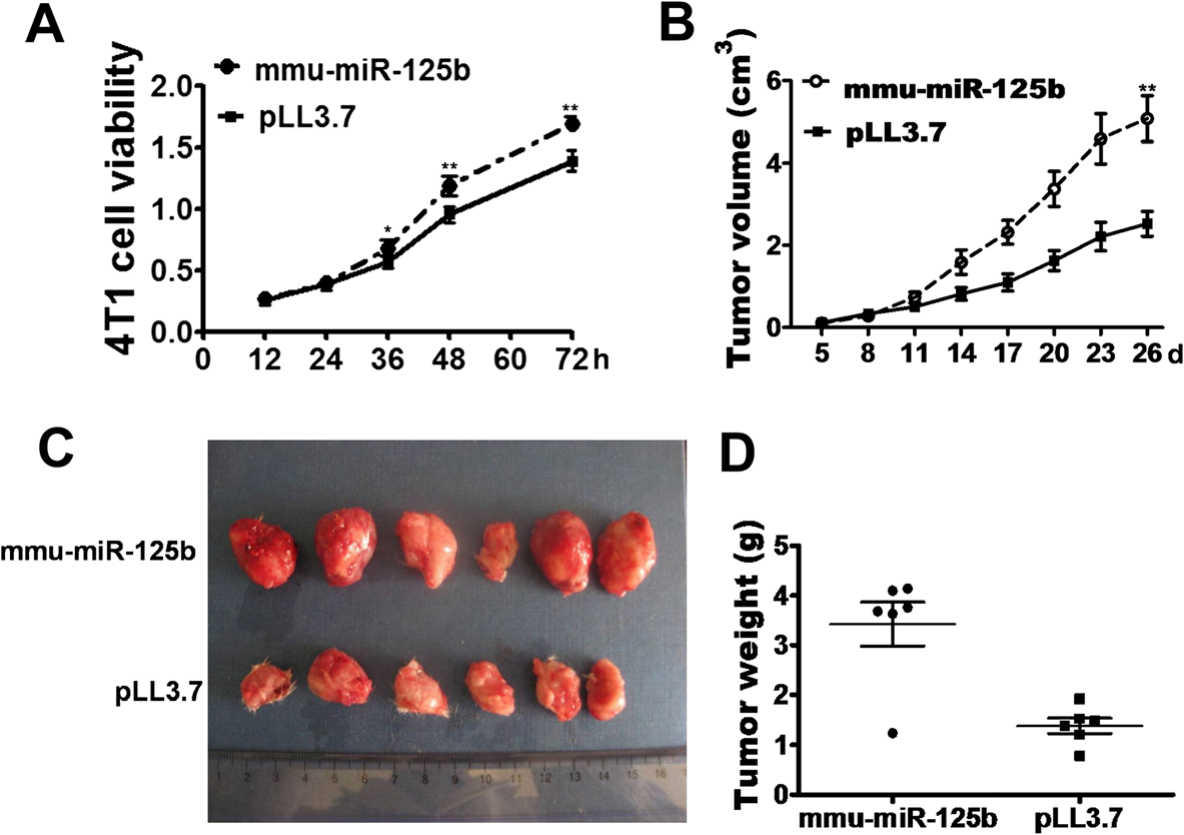
LPS-activated RAW264.7-miR-125b cells promote 4T1 cell proliferation in vitro and in vivo. **a** 4T1 cells were cocultured with either RAW macrophage cells. Briefly, for coculture without cell-cell contact, 1 × 10^5^ LPS-activated RAW264.7-miR-125b or RAW264.7-pLL3.7 cells were seeded in Boyden Transwell inserts. Transwells containing macrophages were then inserted into a 24-well plate and seeded with 3 × 10^5^ 4T1 tumor cells in each well. The cell viability of 4T1 cells was measured with MTS assay at different time points. Each bar represents the mean ± SD (*n* = 3) of three independent experiments. **p < 0.01; *p < 0.05. **b**-**d**. LPS-activated RAW264.7-miR-125b cells promote tumor growth in vivo. 4T1 cells and LPS-activated RAW264.7-miR-125b cells or LPS-activated RAW264.7-pLL3.7 cells were mixed at a ratio of 4:1 and then s.c. co-injected into 4- to 6-week-old BALB/c female mice. After 5 days, tumor length and width were measured with a caliper every 3 days for 3 weeks. The tumor volume at different time points is shown in panels** b** and **c**. Individual tumor weights and the average tumor weight at the experimental endpoint are shown in panels **d**. **p < 0.01; *p < 0.05

One major function of macrophages is to eliminate aberrant cells, such as tumorigenic cells. NO is one of the important molecules that kill tumor cells. To assess the impact of miR-125b overexpression on the proliferation of activated macrophages, we performed a series of ex vivo experiments involving coculture of 4T1 cells with the RAW 264.7 macrophage cell line without cell-cell contact. To test the effect of miR-125b on the growth of the macrophage, MTT assay was conducted to compare the growth rates of the control and over-expressing macrophages. As shown in Additional file 3: Figure S1, there was no obvious difference of growth rate between these two types of macrophages. However, LPS-activated RAW264.7-miR-125b cells, but not control cells (RAW264.7-pLL3.7), significantly promoted the proliferation of cocultured 4T1 cells without cell-cell contact (Fig. 3a), suggesting that macrophages with miR-125b overexpression enhance tumor cell growth.

### LPS-activated RAW264.7-miR-125b cells promote tumor growth in vivo

To further test whether mmu-miR-125b over-expression in RAW264.7 cells affects tumor growth in vivo, LPS-activated RAW264.7-miR-125b or RAW264.7-pLL3.7 control cells and 4T1 cells were mixed at a ratio of 1:4 and then s.c. injected into 4- to 6-week-old BALB/c female mice. Tumor growth was observed for 21 days, and the tumor length and width were measured with a caliper every 3 days. At the end of the experiment, the animals were euthanized, and the tumors were excised and weighed. As shown in Fig. 3, both the volume (Fig. 3b-c) and weight (Fig. 3d) of the tumors derived from LPS-activated RAW264.7-miR-125b cells plus 4T1 cells were much greater than those derived from control RAW264.7 cells plus 4T1 cells These results were consistent with the in vitro data; thus, mmu-miR-125b over-expression in macrophages promotes tumor growth in vivo.

### Mmu-miR-125b inhibits NO production by targeting CCNA2 and eEF2K in macrophages

**Fig. 4:**
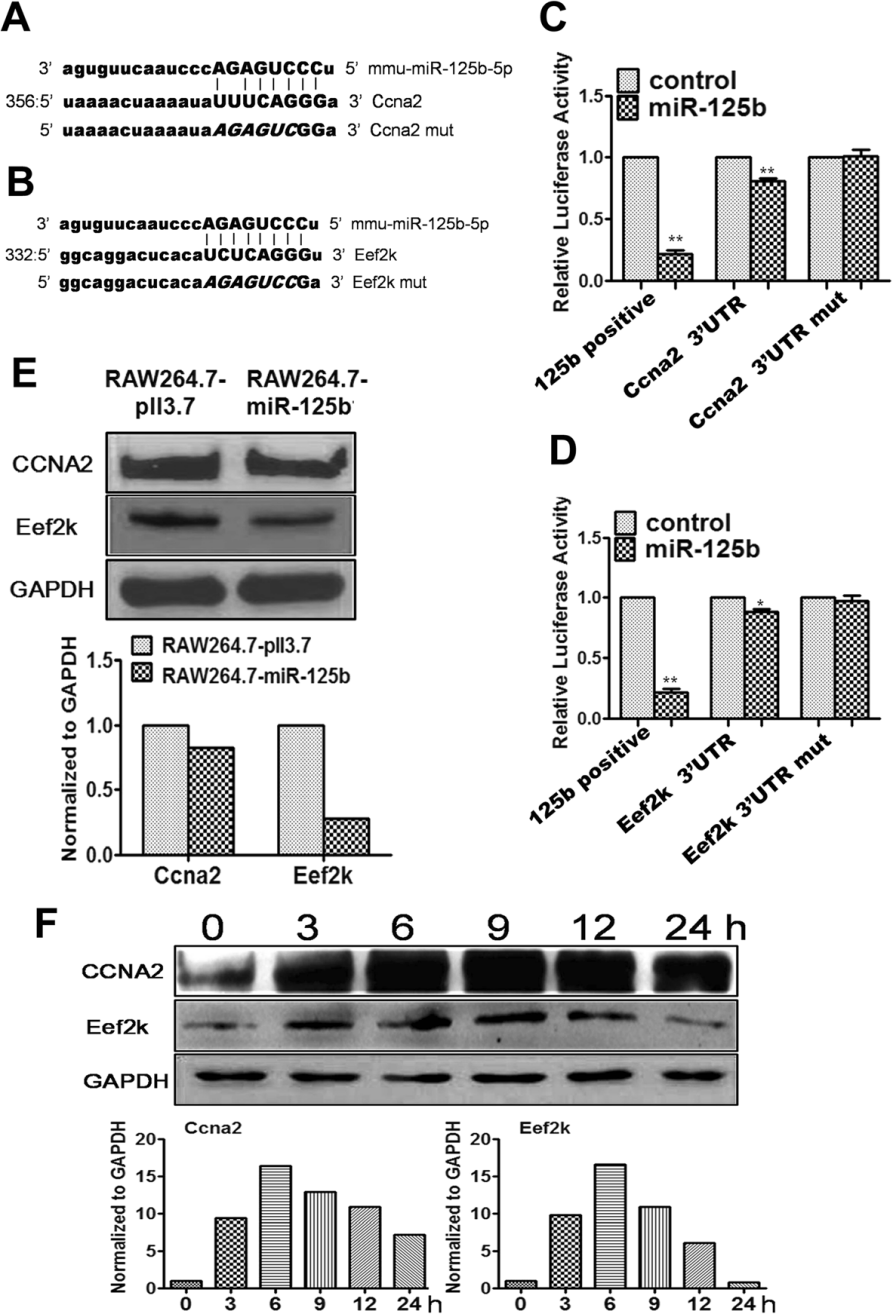
Validation of mmu-miR-125b targets. **a**-**b** Alignment of potential mmu-miR-125b binding sites and mutations in the 3’ UTR of CCNA2 and eEF2K mRNA in mus musculus. **c**-**d** The intact or mutant 3’ UTR of the indicated genes were cloned into the psiCHECK2.2 luciferase reporter vector and then co-transfected with a mmu-miR-125b expression vector (miR-125b) or pLL3.7 (control) into 293T cells. Luciferase activity was analyzed 48 h after transfection using a dual luciferase reporter assay. 125b positive means the psiCHECK2.2 luciferase reporter vector include a sequence totally combined to miR-125b seed sequence. The results are expressed as the relative luciferase activity (firefly/renilla luciferase). The data are presented as the mean ± SD (*n* = 3) of three independent experiments. **e** The protein levels of CCNA2 and eEF2K in 24 h after 1 μg/ml LPS-activated RAW264.7-miR-125b and control cells were determined by western blot; GAPDH served as the loading control. **f** The protein levels of CCNA2 and eEF2K in 1 μg/ml LPS-activated RAW264.7 cells at different time points were determined by western blot; GAPDH served as the loading control. **p < 0.01; *p < 0.05

Usually, miRNAs function by targeting protein-coding genes. Therefore, the direct targets of mmu-miR-125b were investigated. iTRAQ mass spectrometry-based protein detection was performed in RAW264.7 cells transfected with either the mmu-miR-125b over-expression construct or the control. There were 201 differentially expressed proteins (Additional file 4: Table S3) that were decreased more than 25 % compared with the control, suggesting that these 201 genes were probably regulated by mmu-miR-125b. These 201 genes were analyzed using TargetScan (http://www.targetscan.org/mmu_61/) and the microRNA database (http://www.microrna.org/microrna/home.do) to confirm the direct targets of mmu-miR-125b. Bioinformatics analysis of potential mmu-miR-125b binding sites revealed that the 3’ UTR of CCNA2 and eEF2K each harbor a conserved mmu-miR-125b binding site (Fig. 4a-b). To confirm the regulatory interaction, the effects of mmu-miR-125b on eEF2K and CCNA2 reporter genes were evaluated. Luciferase reporter assays confirmed that CCNA2 and eEF2K expression was indeed repressed by mmu-miR-125b via the 3’ UTR (Fig. 4c-d). CCNA2 and eEF2K protein levels were shown to be down-regulated in LPS-activated RAW264.7-miR-125b cells by western blot (Fig. 4e). Mmu-miR-125b is down-regulated in LPS-activated macrophages, and, accordingly, the targets of mmu-miR-125b are expected to be upregulated in LPS-activated macrophages. Through western blotting, we found that eEF2K and CCNA2 were upregulated in LPS-activated macrophages (Fig. 4f). These data indicate that the mmu-miR-125b-mediated inhibition of NO production might occur via targeting eEF2K and CCNA2 in macrophages.

**Fig. 5:**
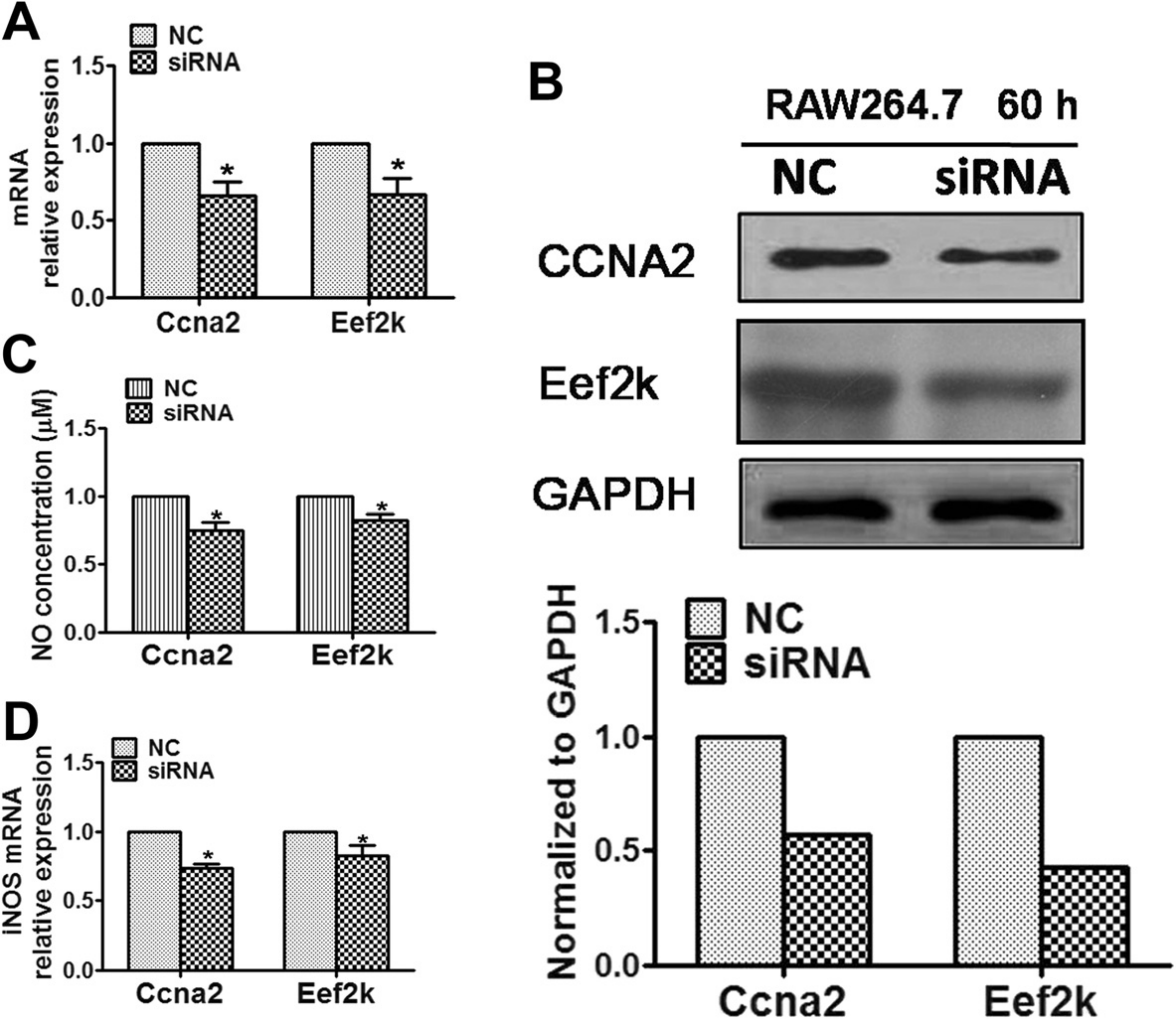
Knockdown of CCNA2 and eEF2K inhibits NO production and iNOS expression in LPS-activated RAW264.7 cells. **a** RAW264.7 cells were transfected with CCNA2 and eEF2K siRNA at a final concentration of 40 nM for 48 h; mRNA levels were determined by qPCR and normalized to β-actin. **b** After 60 h, protein expression was determined by western blot; GAPDH served as the loading control. **c** RAW264.7 cells were transfected with CCNA2 and eEF2K siRNA at a final concentration of 40 nM; after 60 h, the cells were stimulated with 1 μg/ml LPS for 6 h. NO in the supernatant was measured using a nitrate/nitrite assay kit, and the values were normalized to total protein concentration. **d** iNOS mRNA expression was detected by qPCR and normalized to β-actin. The data are presented as the mean ± SD (*n* = 3) of three independent experiments. **p < 0.01; *p < 0.05

The association of eEF2K and CCNA2 with macrophage activation has not been previously reported. Therefore, to confirm the role of eEF2K and CCNA2 in NO production in macrophages, eEF2K and CCNA2 were knocked down in LPS-activated RAW264.7 cells using RNA interference technology (siRNA sequences are provided in Additional file 2: Table S2), and then NO production and iNOS expression were examined. As shown in Fig. 5, knockdown of eEF2K and CCNA2 (Fig. 5a-b) resulted in a significant decrease in NO production (Fig. 5c) and iNOS gene expression (Fig. 5d). Thus, eEF2K and CCNA2 knockdown in RAW264.7 cells mimics the mmu-miR-125b over-expression phenotype. Taken together, our data suggest that eEF2K and CCNA2 are the primary targets of mmu-miR-125b in the regulation of NO production in activated macrophages.

## Discussion

In the present study, we demonstrate that mmu-miR-125b is down-regulated in LPS-activated RAW264.7 cells and peritoneal macrophages and that over-expression of mmu-miR-125b inhibits iNOS expression and NO production in these cells. There have been reports that miR-125b expression decreases in macrophages 3 h after inflammatory stimulation [21, 23]; thus, miR-125b down-regulation may serve as a natural mechanism to promote the inflammatory response.

iNOS induction and NO production are important macrophage functions related to killing NO-sensitive tumors; indeed, tumor cell killing is one of the major functions of macrophages attributed to NO [6, 7, 30]. Therefore, the cytotoxic effects of NO on NO-sensitive cancer cells comprise part of the immune response against tumors [31, 32]. It is well known that TAMs (tumor-associate macrophages) have a reduced capacity to produce anti-tumor molecules, such as NO, TNFα, ROS, and IL-1; instead, TAMs support tumor survival, growth and metastasis and play a pivotal role in tumor angiogenesis and immune evasion [8, 33, 34]. We also found that mmu-miR-125b levels are up-regulated in mouse breast cancer TAMs (data not show). Thus, regulating miR-125b expression might be a potential strategy for influencing macrophage function and eliminating certain cancers.

CCNA2 (NM_009828.2) is critical for the initiation of DNA replication, transcription and cell cycle regulation. CCNA2 has been reported to be a key regulator of cell differentiation, and it can switch the differentiation pathways of human myeloid leukemia K562 cells [35, 36]. eEF2K (NM_007908.4) is a Ca^2+^/calmodulin-dependent protein kinase that regulates JNK (c-jun N-terminal kinase) and NF-κB (nuclear factor-kappa B) p65 phosphorylation as well as reactive oxygen species (ROS) production and also affects the development of hypertension [37, 38]. We demonstrated that the knockdown of eEF2K and CCNA2 significantly decreases NO production and iNOS gene expression in activated macrophages. The association of eEF2K and CCNA2 with macrophage activation has not been previously reported. However, the other functions and mechanisms of action of eEF2K and CCNA2 in activated macrophages need to be further clarified.

The level of iNOS expression and NO production suppression suggests that mmu-miR-125b is a contributing albeit relatively modest impact on the regulation of the NO production pathway. The biological process is complex and is regulated by signal pathway network. There might be multiple factors to regulate the NO production pathway and mmu-miR-125b might be one of molecules in this complex network. Similarly, it seems that the modest effect of si-RNA knockdown of eEF2K and CCNA2 transcripts on NO production and iNOS expression also argues that the contributions of mmu-miR-125b and its target genes. Numerous studies revealed a one-to-one relationship between miRNA and its target gene. However, one miRNA may regulate many genes as its targets, while one gene may be targeted by many miRNAs. So the effect of miRNA regulation on mRNA and protein levels is usually quite modest and associated phenotypes are often weak or subtle. It is now becoming clear that complex regulatory networks between miRNAs and their gene targets are actually common mechanisms that have evolved in gene regulation. Therefore, our data suggest that mmu-miR-125b decreases NO production in activated macrophages partially by suppressing eEF2K and CCNA2 expression because of the complex regulatory networks.

LPS was used to activate macrophages in this study because it was a stimulator of M1 macrophages which could secrete high amounts of pro-inflammatory mediators to kill invading pathogens or tumor cells. It is not typical experimental designs to directly test mechanistic hypothesis. But the results obtained from LPS activated macrophages suggested the possibility of reverse tumor-polarized tumor associated macrophages phenotype and re-educate them to kill tumor cells by M1 stimulators.

## Conclusions

We have shown in this study that increased mmu-miR-125b expression in macrophages promotes 4T1 cell growth in vitro and in vivo. Therefore, knockdown of miR-125b expression in macrophages in the tumor microenvironment may be a useful strategy for the treatment of certain cancers. These findings may extend our understanding of the function of miR-125b in regulating macrophage activation and the immune response.

## Additional files

Additional file 1: Table S1. Primers used in this study. (DOC 32 kb) Additional file 2: Table S2. Ccna2 and Eef2k siRNA sequences. (DOC 30 kb) Additional file 3: Figure S1. Proliferation of RAW264.7 cells stably overexpressing mmu-miR-125b was assessed by MTS assay. (TIFF 434 kb) Additional file 4: Table S3. 201 differentially expressed proteins that decreased more than 25 % compared to the controls were detected by iTRAQ mass spectrometry. (XLSX 25 kb)

## Abbreviations

miRNAs (miR): microRNAs
mRNA: messenger RNA
3’-UTR: 3’ untranslated region
NC: negative control
RNAi: RNA interference
siRNA: small interfering RNA
DMEM: Dulbecco’s modified Eagle’s medium
FBS: fetal bovine serum
TBS: Tris-buffered saline
PBS: phosphate-buffered saline
CCNA2: cyclin A2
eEF2K: eukaryotic elongation factor 2 kinase
iNOS: inducible nitric oxide synthase
LPS: lipopolysaccharide
qPCR: quantitative real-time PCR
NO: nitric oxide
PMs: peritoneal macrophages
TLRs: Toll-like receptors
