# Supplementary material for: Mmu-miR-125b overexpression suppresses NO production in activated macrophages by targeting eEF2K and CCNA2
Source: BMC Cancer. 2016 Mar 28;16:252. doi: 10.1186/s12885-016-2288-z (PMC4809031; doi:10.1186/s12885-016-2288-z)
Supplement: Additional file 1: Table S1. — Primers used in this study. (DOC 32 kb) [file 12885_2016_2288_MOESM1_ESM.doc]

**Table S1: Primers used in the experiment**

| Gene | Forward (5’-3’) | Reverse (5’-3’) |
| --- | --- | --- |
| mmu-miR-125b | GGTCCCTGAGACCCTAAC | CAGTGCGTGTCGTGGAGT |
| U6 | GCTTCGGCAGCACATATACTAAAAT | CGCTTCACGAATTTGCGTGTCAT |
| pre-miR-125b | AAAAGTTAACCTGTGTGTTTCAATTAGTATTTAGAAGATTAAAA | CCGCTCGAGTTAGAAAGAAAACCATTGTTCTTTGCG |
| iNOS | GTTCTCAGCCCAACAATACAAGA | GTGGACGGGTCGATGTCAC |
| Ccna2 | ACTGCTCGAGGTACATGTGTCTATTTGGAT | AGTCACTAGTCTTACAAGCTGAACTTCTTG |
| Eef2k | ACTGCTCGAGTTCCGTTTTCTCACTCTTGA | AGTCACTAGTTCAGAGGTAGTAAATACGCA |
| β-actin | CATGTACGTTGCTATCCAGGC | CTCCTTAATGTCACGCACGAT |
| oligo dT | TTTTTTTTTTTTTTT | |
| U6 RT | CGCTTCACGAATTTGCGTGTCAT | |
| 125bRT | GTCGTATCCAGTGCGTGTCGTGGAGTCGGCAATTGCACTGGATACGACTCACAA | |
