# Supplementary material for: Mmu-miR-125b overexpression suppresses NO production in activated macrophages by targeting eEF2K and CCNA2
Source: BMC Cancer. 2016 Mar 28;16:252. doi: 10.1186/s12885-016-2288-z (PMC4809031; doi:10.1186/s12885-016-2288-z)
Supplement: Additional file 2: Table S2. — Ccna2 and Eef2k siRNA sequences. (DOC 30 kb) [file 12885_2016_2288_MOESM2_ESM.doc]

**Table S2: SiRNA sequence of Ccna2 and Eef2k used in the experiment**

| Gene | siRNA sense sequence（5'-3'） | siRNA antisense sequence（5'-3'） |
| --- | --- | --- |
| Ccna2-mus-1059 | GGCUGCACCAACAGUAAAUTT | AUUUACUGUUGGUGCAGCCTT |
| Ccna2-mus-801 | GGUGGGAGAAGAAUAUAAATT | UUUAUAUUCUUCUCCCACCTT |
| Ccna2-mus-1270 | GCACAACAGACUGGAUAUATT | UAUAUCCAGUCUGUUGUGCTT |
| Eef2k-mus-2437 | CAGCAAUGGAAGCCAUGAATT | UUCAUGGCUUCCAUUGCUGTT |
| Eef2k-mus-1042 | GCAAGUACAUCAAGUACAATT | UUGUACUUGAUGUACUUGCTT |
| Eef2k-mus-952 | CCAAGCAGGUGGAUAUCAUTT | AUGAUAUCCACCUGCUUGGTT |
