# Supplementary figures and images for: Mmu-miR-125b overexpression suppresses NO production in activated macrophages by targeting eEF2K and CCNA2
Source: BMC Cancer. 2016 Mar 28;16:252. doi: 10.1186/s12885-016-2288-z (PMC4809031; doi:10.1186/s12885-016-2288-z)

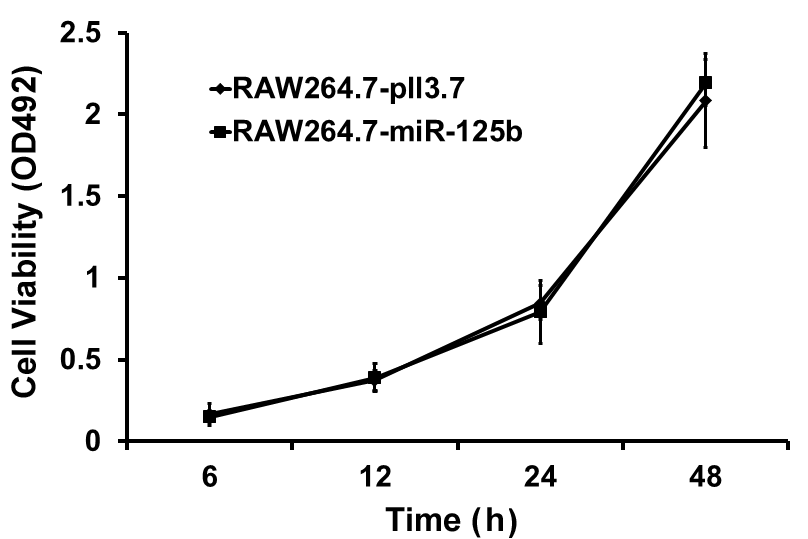

Supplement: Additional file 3: Figure S1. — Proliferation of RAW264.7 cells stably overexpressing mmu-miR-125b was assessed by MTS assay. (TIFF 434 kb) [file 12885_2016_2288_MOESM3_ESM.tiff]
